# PReS-FINAL-2326: No correlation between anti-drug antibodies and pharmacokinetics, efficacy or safety of Anakinra (Kineret^®^) in patients with severe CAPS

**DOI:** 10.1186/1546-0096-11-S2-P316

**Published:** 2013-12-05

**Authors:** M Wikén, P Gozzi, G Andersson, H Olivecrona, M Aldén Raboisson, T Kullenberg, M Leinonen, B Hallén

**Affiliations:** 1 Swedish Orphan Biovitrum AB, Stockholm, Sweden

## Introduction

Anakinra is a recombinant, non-glycosylated form of the human IL-1 receptor antagonist, which recently was approved in US for neonatal onset multisystem inflammatory disease (NOMID), the most severe form of the Cryopyrin-Associated Periodic Syndromes (CAPS). This autoinflammatory disease can be effectively controlled by daily subcutaneous administrations of Kineret[1-3]. The recommended initial dose is 1-2 mg/kg/day and the average maintenance dose is 3-4 mg/kg/day.

## Objectives

To evaluate if anti-drug antibodies (ADAs) correlate with outcome measures in severe CAPS patients on Kineret.

## Methods

Forty patients treated up to 5 years and with pre- and post-baseline antibody data were included in long-term evaluations of ADA impact on pharmacokinetic parameters (dose-normalized maximum serum concentration, C_max_, and area under the serum concentration time curve, AUC_0-24h_), body-weight adjusted Kineret dose, change in Diary Symptom Sum Score (DSSS, i.e. sum score for fever, rash, joint pain, vomiting, and headache), change in C-reactive protein (hs-CRP) levels, and treatment emergent adverse events (AEs).

ADAs were assessed with a bridging format immunoassay applying Meso Scale Discovery Electrochemiluminescence (MSD-ECL) technology. Data from pre-dose samples were used to calculate a cut point with a false positive rate of 5%. Screened positive samples were further confirmed by competitive inhibition.

For statistical analyses the patients were classified based on the presence of ADAs.

## Results

No patient had ADAs at baseline, while 82.5% of the patients exhibited ADAs at least once during the study. The exposure to Kineret was 73.8 patient-years when ADAs were not present and 82.2 patient-years when ADAs were present. The daily dose of Kineret at Month 36 [mean(SD)] for patients with ADAs not present was 2.7(0.6) mg/kg and for patients with ADAs present 3.1(0.8) mg/kg. The following estimates for the ADA negative and ADA positive patient groups, respectively, were obtained: dose normalized C_max _1164(554) *vs*. 941(406) ng/mL/mg/kg, dose normalized AUC 15.0(6.5) *vs*. 12.6(5.8) μg·h/mL/mg/kg, change in hs-CRP from baseline -53(41) *vs*. -60(34) mg/L, and change in DSSS from baseline -2.5(1.8) *vs*. -2.7(1.4). DSSS decreased significantly within a few days in all patients with sustained effect and continuous suppression of inflammatory serum markers. No trend by antibody status was seen for the rate of AEs related to allergies, injection site reactions or symptoms of CAPS.

## Conclusion

The majority of the severe CAPS patients on Kineret developed transient or persistent ADAs. No correlation was seen between the presence of ADAs and Kineret dose, PK parameters, efficacy outcomes or AEs.

## Disclosure of interest

M. Wikén Employee of: Employee of Swedish Orphan Biovitrum AB, P. Gozzi Shareholder of: Shareholder of Swedish Orphan Biovitrum AB, Employee of: Employee of Swedish Orphan Biovitrum AB, G. Andersson Shareholder of: Shareholder of Swedish Orphan Biovitrum AB, Employee of: Employee of Swedish Orphan Biovitrum AB, H. Olivecrona Employee of: Employee of Swedish Orphan Biovitrum AB, M. Aldén Raboisson Shareholder of: Shareholder of Swedish Orphan Biovitrum AB, Employee of: Employee of Swedish Orphan Biovitrum AB, T. Kullenberg Employee of: Employee of Swedish Orphan Biovitrum AB, M. Leinonen: None Declared, B. Hallén Shareholder of: Shareholder of Swedish Orphan Biovitrum AB, Employee of: Employee of Swedish Orphan Biovitrum AB.
